# Treatment for ovarian clear cell carcinoma with combined inhibition of WEE1 and ATR

**DOI:** 10.1186/s13048-023-01160-y

**Published:** 2023-04-22

**Authors:** Wenwen Chien, Jeffrey W. Tyner, Sigal Gery, Yueyuan Zheng, Li-Yan Li, Mohan Shankar Gopinatha Pillai, Chehyun Nam, Neil A. Bhowmick, De-Chen Lin, H. Phillip Koeffler

**Affiliations:** 1 Department of Medicine, Samuel Oschin Comprehensive Cancer Institute, Cedars-Sinai Medical Center, 8700 Beverly Boulevard, Los Angeles, CA 90048 USA; 2 Knight Cancer Institute, Oregon Health & Science University, Oregon Health and Science University, 2720 S.W. Moody Avenue, Portland, OR 97201 USA; 3 Clinical Big Data Research Center, Scientific Research Center, The Seventh Affiliated Hospital of Sun Yat-Sen University, Shenzhen, 518107 P. R. China; 4 The Key Laboratory of Molecular Biology for High Cancer Incidence Coastal Chaoshan Area, Shantou University Medical College, Shantou, Guandong Province P. R. China; 5 Center for Craniofacial Molecular Biology, University of Southern California, Los Angeles, CA 90089 USA; 6 Department of Hematology-Oncology, National University Cancer Institute of Singapore, National University Hospital, Singapore, 119074 Singapore

**Keywords:** Ovarian clear cell carcinoma, ATR, WEE1

## Abstract

**Background:**

Standard platinum-based therapy for ovarian cancer is inefficient against ovarian clear cell carcinoma (OCCC). OCCC is a distinct subtype of epithelial ovarian cancer. OCCC constitutes 25% of ovarian cancers in East Asia (Japan, Korea, China, Singapore) and 6–10% in Europe and North America. The cancer is characterized by frequent inactivation of ARID1A and 10% of cases of endometriosis progression to OCCC. The aim of this study was to identify drugs that are either FDA-approved or in clinical trials for the treatment of OCCC.

**Results:**

High throughput screening of 166 compounds that are either FDA-approved, in clinical trials or are in pre-clinical studies identified several cytotoxic compounds against OCCC. ARID1A knockdown cells were more sensitive to inhibitors of either mTOR (PP242), dual mTOR/PI3K (GDC0941), ATR (AZD6738) or MDM2 (RG7388) compared to control cells. Also, compounds targeting BH3 domain (AZD4320) and SRC (AZD0530) displayed preferential cytotoxicity against ARID1A mutant cell lines. In addition, WEE1 inhibitor (AZD1775) showed broad cytotoxicity toward OCCC cell lines, irrespective of ARID1A status.

**Conclusions:**

In a selection of 166 compounds we showed that inhibitors of ATR and WEE1 were cytotoxic against a panel of OCCC cell lines. These two drugs are already in other clinical trials, making them ideal candidates for treatment of OCCC.

**Supplementary Information:**

The online version contains supplementary material available at 10.1186/s13048-023-01160-y.

## Background

Ovarian clear cell carcinoma (OCCC) patients show low response rates (10–25%) to standard ovarian cancer therapy (carboplatin and paclitaxel). This therapy is effective (~ 80% response rate) in other subtypes (high-grade serous, endometrioid) of ovarian cancer [1, 2]. When adjusted for stage, OCCC has the worst prognosis [3]. Moreover, patients with either late stage or progressive disease of OCCC have poor 5-year survival rate (13%) [4]. OCCC constitutes ~ 6% of ovarian cancer in North America and Europe [3] and has high prevalence (25%) in East Asia (Japan, Korea, China, Singapore) [5–7]. The most frequently mutated gene in OCCC is ARID1A (AT-rich interactive domain-containing protein 1 A) occurring in 50% of patients [8–10]. Most of ARID1A mutations result in loss of its protein expression [9, 11]. Several potential therapeutic targets have been identified for OCCC [10, 12, 13]. In addition, synthetic lethality targeting ARID1A mutation has identified several inhibitors [14, 15] including those targeting EZH2, BET, and HDAC6 [16–18] which have led to several in clinical trials (e.g. NCT03348631, NCT03297424). However, these treatment strategies have not been successfully translated to the clinic. Alternative treatment and discovery of novel therapeutic targets are urgently needed to improve OCCC patient outcomes.

We established ARID1A isogenic OCCC cell lines and performed high throughput drug screening using 166 compounds (FDA-approved, or either in clinical trials or pre-clinical studies). We identified 7 inhibitors of OCCC targeting WEE1 (AZD1775), mTOR (PP242), dual mTOR/PI3K (GDC0941), ATR (AZD6738), MDM2 (RG7388), BH3 domain (AZD4320) and SRC (AZD0530). We found that the combination of WEE1 (AZD1775) and ATR (AZD6738) inhibitors synergistically killed the OCCC cells. These two drugs are in clinical trials for other cancer-types, making them ideal candidates for treatment of OCCC.

## Methods

### High-throughput drug screening

OVCA429 OCCC cell line [16, 17, 19–24] was used to establish ARID1A isogenic cells (WTC: OVCA429-shC; KD: OVCA429-sh1) and were screened against a panel of 166 small-molecule inhibitors (single agent or combination of two) (Supplementary Table 1) as previously described [25]. These drugs are inhibitors targeting several tyrosine and non-tryosine kinase pathways; MAPKs, MTOR-PI3K-AKT, RAF, AMPK, ATM, ATR, Aurora kinases, cyclin-dependent kinases (CDKs), calcium/calmodulin-dependent protein kinase (CAMKs), serine/threonine protein kinase 3 (GSK3), protein kinase C, polo-like kinase 1 (PLK1). Additionally, the panel contains drugs with anti-tumor activity against BCL2 family members, Bromodomain and Extra-Terminal Domain (BET) family, as well as against Hedgehog, HSP90, NOTCH, proteasome, HDAC, STAT3, and WNT/β-catenin. Drugs were purchased from LC Laboratories and Selleck Chemicals. Each drug was prepared and diluted in a series of 3-fold dilutions to a final range of 10 µM to 0.137 nM. Briefly, 10,000 cells were treated with drugs in 384-well plates for 72 h; cell viability was determined using MTS reagent (CellTiter96 AQeous One, Promega) after normalization with untreated control cells.

### Western blot analysis

Cells were harvested in the presence of lysis buffer (25 mM Tris•HCl pH 7.4, 150 mM NaCl, 1% NP-40, 1 mM EDTA) plus protease inhibitor cOmplete cocktail (Sigma). Protein lysates were separated by SDS polyacrylamide gel electrophoresis followed by Western blotting with different antibodies. Expression levels of individual protein were quantified using ImageJ [26]. Representative Western blots were shown from 2 independent experiments. Two-tailed t test was performed [Compared to control: * (p < 0.05), ** (p < 0.01), *** (p < 0.001), **** (p < 0.0001); Combination treatment compared to single agent: # (p < 0.05), ## (p < 0.01), ### (p < 0.001)].

### Real-time PCR

RNA extraction from cells were performed by using RNeasy mini kit (Qiagen). After digestion with DNase (ThermoFisher Scientific), cDNA was synthesized using MAXIMA H(-) MASTERMIX (ThermoFisher Scientific). Real-time PCR was performed using POWRUP SYBR MASTER MIX (ThermoFisher Scientific) in CFX96 Real-time system (Bio-Rad).

### Cell Culture

Human OCCC cell lines (WT: ARID1A wild type: JHOC5, ES2, OVCA429, RMG1; Mut: ARID1A mutant: OVTOKO, KOC7C, TAYA, JHOC9, RMG5) were from in-house collection of ovarian cancer cell lines purchased or acquired from various sources described in Supplementary Table 5 [27–29]. Cell lines were maintained in appropriate culture media (DMEM or RMPI-1640) with 10% FBS. Expression levels of ARID1A were examined by Western blot analysis (Supplementary Fig. 1A). Hereafter for comparison between ARID1A wild type and mutant cell lines, they are referred as WT and Mut.

### shRNA knockdown of ARID1A

Lentiviral vector pLKO.1 was used to select stable silencing (shRNA) of ARID1A in 3 ARID1A wild type cell lines (ES2, JHOC5, OVCA429) by puromycin. Two shRNA target sequences for ARID1A were Sh1 (GAAAGCGAGGGCCCCGCCGT) and sh5 (GCTTCGGGCAACCCTACGGC) as well as control scrambled shRNA (shC) (GAACCTATTCCCGCAATCTAA). Isogenic silencing of ARID1A in OVCA429, ES2, JHOC5 was verified by real-time PCR and Western blots (Supplementary Fig. 1B). Hereafter for comparison between ARID1A wildtype control to isogenic knockdown cells, they are referred as WTC and KD.

### Second round drug screening and validation

The hit drugs selected from high-throughput screening were further screened and validated between 10 µM to 10 nM. Cells (2,500/well) were treated with drugs in 96-well plates for 72 h; and cell viability was measured using MTT (3-(4,5-dimethylthiazol-2-yl)-2,5-diphenyltetrazolium bromide). All experiments were performed three times in triplicates. IC50 values were generated using Graphpad Prism software.

### Statistics

Data were analyzed by either Student’s t-test or two-way Anova. Generation of hierarchical clustering of heatmap was performed using ClustVis [30].

### Soft agar colony formation assay

OCCC (2,000–3,000 cells) were grown for 14 days in top layer of 0.3% agar with a bottom layer of 0.6% agar in 24-well plates. Drugs were mixed with top layer of soft agar at day 1. Colonies were fixed with formaldehyde and stained with crystal violet. Each assay was performed in triplicates and repeated once. ImageJ2 was used to quantify number of colonies [26, 31]. Representative results are shown from 2 independent experiments. Two-tailed t test was performed.

### Public databases and web application

GDSC (The Genomics of Drug Sensitivity of Cancer database) contains drug response data and genomic markers of sensitivity from ~ 1,000 human cancer cell lines screened with 180 to 400 compounds [32]. This database contains 7 OCCC cell lines (4 Mut: TOV21G, OVTOKO, OVISE, OCI314; 3 WT: RMG1, ES2, EFO21). A total of 32/166 of the compounds were included in data set GDSC1 and 15/166 in data set GDSC2. DrugComDB contain dose-response data from a combination of 2,877 drugs in 124 human cancer cell lines [33]. It includes 1 OCCC cell line (ES2). CompuSyn was used to calculate combination index (CI) for drug combinations [34]. CI values: < 1, synergistic; = 1, additive; > 1, antagonistic. SynergyFinder was used to analyze synergy scores of drug combinations [35, 36]. Dose-responses were calculated; and interaction between two drugs were analyzed by SynergyFinder 2.0 using HSA (Highest Single Agent) model. HSA model states that the expected combination effect equals to the higher effect of individual drugs. Likelihood between two drugs being: Scores < -10, likely antagonistic; -10 < score < 10, likely additive; score > 10, likely synergistic. Dose-response matrices were generated using LL4 curve fitting, and outliers deleted. Synergy maps and scores were generated based on HSA model.

### Cell cycle and apoptosis analysis

Propidium iodide-stained cells was used to measure DNA contents after drug treatment. Briefly, after drug treatment, cells were fixed with ethanol, treated with RNaseA, and stained with propidium iodide. APC Annexin V Apoptosis Detection Kit (Biolegend) was used to identify apoptotic cells following manufacturer’s instructions. Data were acquired on LSRII flow cytometer and analyzed with FlowJo software. Representative results are shown from 2 independent experiments. Two-tailed t test was performed. *, p < 0.05, compared to control; #, p < 0.05 combination treatment compared to single agent.

## Results

### High-throughput drug screening

**Fig. 1:**
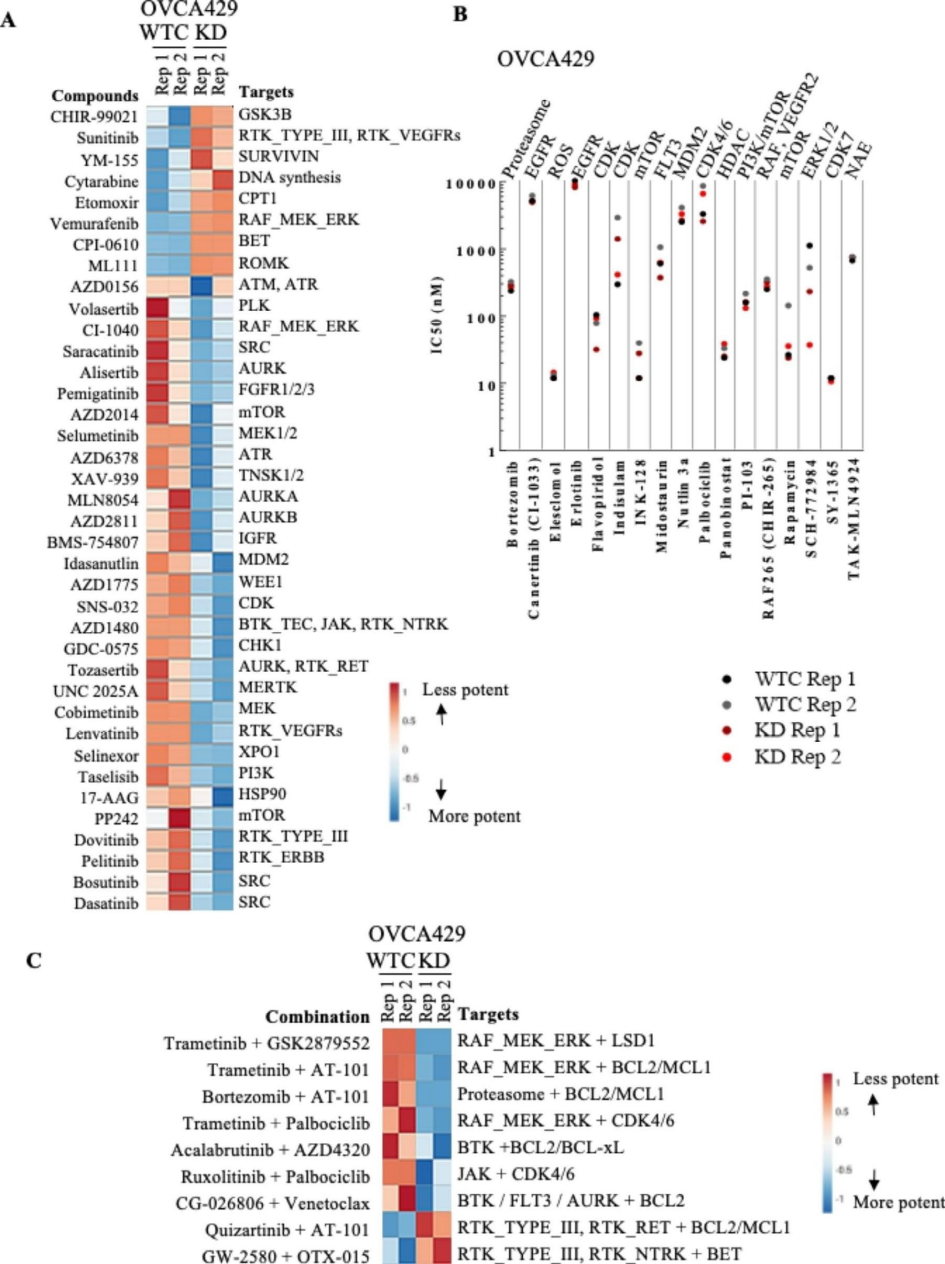
Sensitivity of small molecule inhibitors of OCCC cell lines with silencing of ARID1A. ARID1A isogenic OVCA429 OCCC cell lines were treated with drugs for 72 h with 2 replicates (Rep 1, Rep 2) each from KD (ARID1A shRNA knockdown) versus WTC (wildtype control). (A) The 38 small molecule inhibitors showed significantly different cytotoxicity when comparing KD to WTC. IC50 values (0–10,000 nM, Supplementary Table 2) for each inhibitor are expressed in a clustered heatmap. (B) IC50 of 18 compounds with cytotoxicity toward both KD and WTC are presented in dot plot fashion. (C) Significant differential response to 9 different combination treatment (KD vs. WTC). Red, higher IC50; Blue, lower IC50.

To identify potential new drugs against OCCC, high-throughput screening of 166 small molecules was performed on the ARID1A isogenic OCCC cell line OVCA429: wild type control (WTC) and ARID1A knockdown (KD). Thirty-eight compounds showed significantly (*p* < 0.05) different cytotoxicity between KD and WTC (Fig. 1A, Supplementary Table 2). WTC cells were more sensitive to 8 compounds including sunitinib, cytarabine, vemurafenib, and inhibitors to GSK3B, SURVIVIN, or BET. KD cells were more sensitive to 30 compounds targeting either ATM, ATR, SRC, MDM2, XPO1, WEE1, CDK, or Aurora kinases and several receptor tyrosine kinases including ERBB, VEGFRs, IGFRs. Another 18 compounds exhibited similar cytotoxicity toward both KD and WTC cells including those targeting proteasome, CDK, EGFR, mTOR, dual PI3K/mTOR and HDAC (Fig. 1B, Supplementary Table 3). Nine combinations of 2 drugs showed significantly different potency comparing KD and WTC cells (*p* < 0.05, Fig. 1C, Supplementary Table 4). WTC cells were more sensitive to combination of a BET inhibitor with a multi-target kinase inhibitor quizartinib. KD cells were more sensitive to CDK4/6 inhibitor in combination with JAK inhibitor ruxolitinib or MEK inhibitor trametinib. BCL2 inhibitors with different combination of kinases were effective in both WTC and KD but co-treatment with BCL2 inhibitor AT-101 and bortezomib produced higher efficacy toward KD cells.

### Validation of hits

**Fig. 2:**
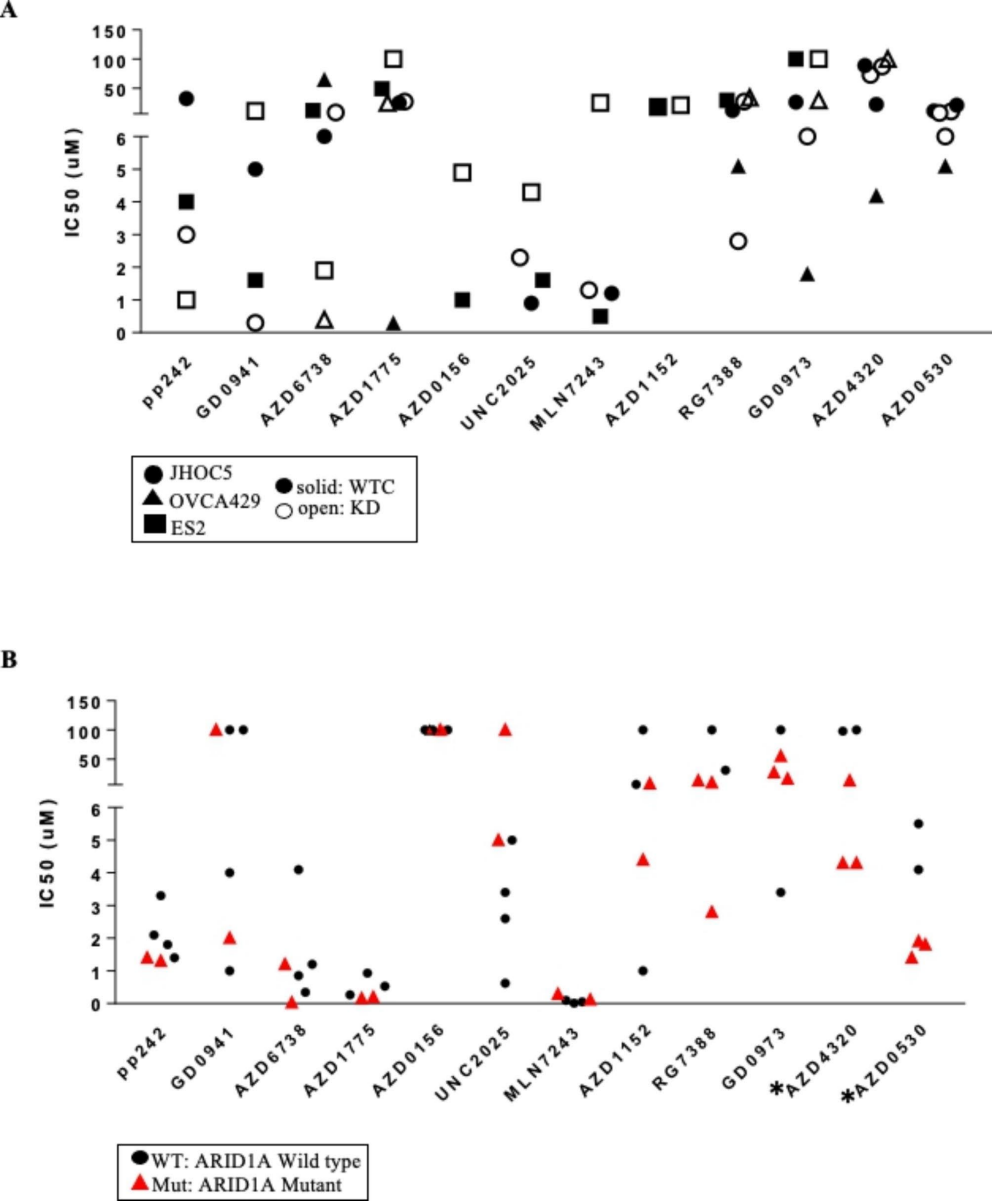
IC50s in OCCC cell lines. (A) Summary of IC50 values in 3 sets of ARID1A isogenic cell lines (JHOC5, ES2, OVCA429; KD: ARID1A shRNA knockdown; WTC: wildtype control). Solid shapes, WTC; Open shapes, KD. (B) Summary of IC50 values in OCCC cell lines (WT: JHOC5, ES2, OVCA429, RMG1; Mut: OVTOKO, KOC7C). Values of IC50 were calculated from dose curves (Supplementary. Figure 2). Circles, WT; Triangles, Mut; *: p < 0.05

Three pairs of ARID1A isogenic OCCC cell lines (OVCA429, ES2, JHOC5) (Supplementary Fig. 1B) were used to validate hits identified by high-throughput screens. To improve accuracies, 12 compounds were selected based on IC50 values and AUC (areas under curve). These drugs were inhibitors targeting mTOR (PP242), dual mTOR/PI3K (GDC0941), ATR (AZD6738), WEE1 (AZD1775), ATM (AZD0156), MERTK (UNC2025), UAE (MLN7243), AURKB (AZD1152), MDM2 (RG7388), MEK1 (GD0973), BH3 (AZD4320), and SRC (AZD0530). From the 12 compounds selected, we identified 4 drugs targeting mTOR (pp242), dual mTOR/PI3K (GDC0942), MDM2 (RG7388) and ATR (AZD6738) were more potent in at least one ARID1A KD cell line versus the rest of 8 drugs in their potency between KD and WTC cells (Fig. 2A).

In addition to isogenic cell lines, drug potency of these 12 compounds were also examined in additional 6 OCCC cell lines (2 Mut: OVTOKO, KOC7C; 4 WT: ES2, JHOC5, OVCA429, RMG1) (Supplementary Fig. 2). Compounds targeting either BH3 domain (AZD4320) or SRC (AZD0530) displayed preferential cytotoxicity with lower IC50 values against ARID1A mutant cell lines (Fig. 2B). PP242 (mTOR), AZD6738 (ATR), AZD1775 (WEE1) also exhibited anti-proliferative activity at < 5 µM in OCCC cell lines (Fig. 2B). Four compounds (AZD6738, AZD1775, GDC0941, AZD0530) of the 12 hits are included in GDSC [32]. In GDSC database, several of the OCCC cell lines had lower IC50 than the geometric IC50 calculated from all the cell lines examined in GDSC indicating these 4 drugs are active in OCCC cell lines (Supplementary Fig. 3). This verifies our observations that these 4 drugs: AZD6738 (ATR inhibitor), AZD1775 (WEE1 inhibitor), GDC0941 (dual mTOR/PI3K inhibitor), AZD0530 (SRC) had anti-proliferative activities in OCCC cell lines.

### AZD6738 (ATR inhibitor) and AZD1775 (WEE1 inhibitor) combination in OCCC

**Fig. 3:**
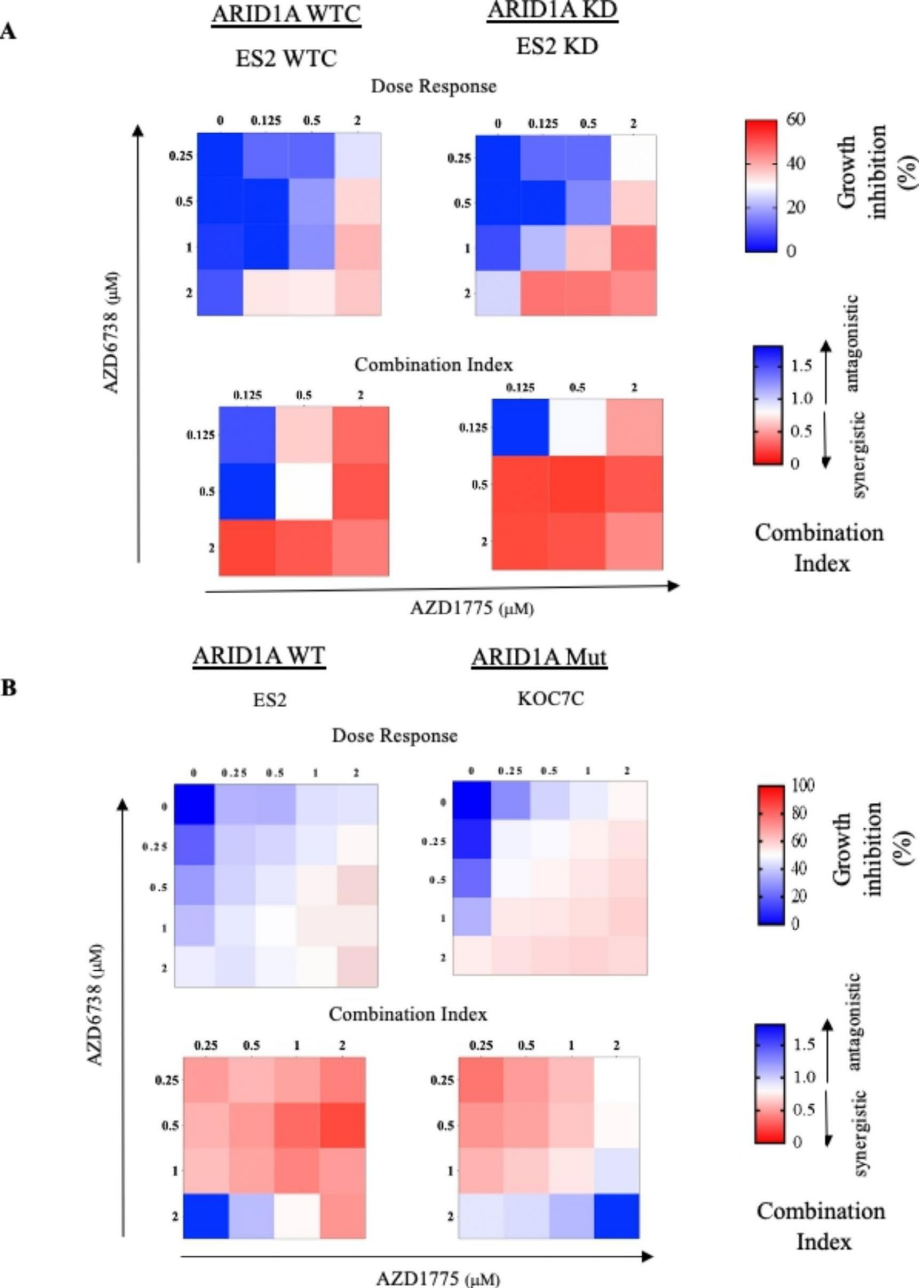
Combination treatment with AZD1775 and AZD6738 in OCCC cell lines. (A) OCCC cell lines (WT: ARID1A wild type, ES2; Mut: ARID1A mutant, KOC7C) were treated with AZD1775 or AZD6738 alone and combination of both at 0, 0.25, 0.5, 1, 2 µM (< IC50) for 72 h. Dose-response matrices and CI (combination index) heatmap between the interaction of two drugs are shown. Dose-response matrices: Red; 100% growth inhibition; White; 50%; Blue, 0%. CI’s were analyzed by CompuSyn as described in Methods. CI values: < 1, synergistic; =1, additive; > 1, antagonistic. CI < 0.3, strong synergy. (B) ARID1A isogenic ES2 OCCC cell lines (WTC : wildtype control; KD: ARID1A shRNA knockdown). Also see Supplementary Fig. 4

Both ATR and WEE1 play important roles in response to DNA damage [37, 38]. Combination of inhibitors to ATR (AZD6738) and WEE1 (AZD1775) was evaluated in 4 OCCC cell lines (3 WT: ES2, OVCA429, RMG1; 3 Mut: KOC7C, OVTOKO, TAYA) and 2 pairs of ARID1A isogeneic cell lines (KD vs. WTC in ES2 and JHOC5). Combination index (CI) was used to analyze for synergism. In isogenic cell models (ES2, JHOC5) with defect in ARID1A, drug combination caused synergistic effect in some combinations (Fig. 3A, Supplementary Fig. 4A) in both WTC and KD cell lines. Majority of combinations also had CI value below 1 in both WT and Mut cell lines suggesting the combination of an ATR inhibitor (AZD6738) with an inhibitor of WEE1 (AZD1775) synergistically inhibited cell growth of OCCC cell lines (Fig. 3B, Supplementary Fig. 4B). Similar results were found when combination effects were analyzed with a different scoring system (SynergyFinder) (Supplementary Fig. 5). Nevertheless, heatmaps of dose response indicated WT cell lines were more sensitive to combination treatment than Mut cell lines (Fig. 3B, Supplementary Fig. 4B).

**Fig. 4:**
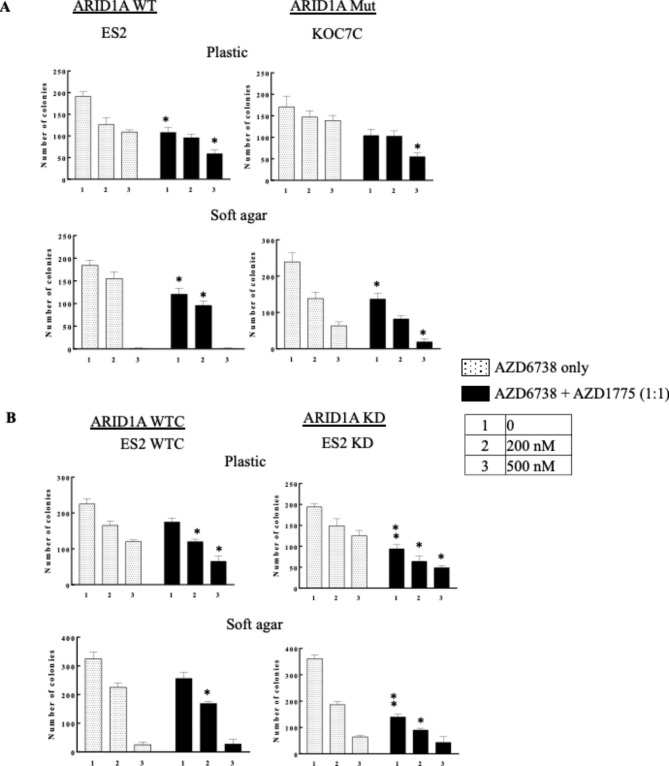
AZD1775 and AZD6738 inhibited cell growth in OCCC cell lines. OCCC cell lines were cultured on plastic or soft agar and treated with drugs for 14 days. Colony formation was fixed, stained, and number of colonies was counted. (A) WT (ES2) and Mut (KOC7C) cell lines. (B) ARID1A isogenic cell lines (ES2 WTC, ES2 KD). (Two-tailed t test of combination to single agent: *, p < 0.05; *, p < 0.01, n = 2)

Colony formation assay showed decrease in number of colonies when OCCC cell lines were treated with AZD6738 (Fig. 4A, plastic, WT: ES2; Mut: KOC7C; Fig. 4B, plastic ES2: WTC and KD). Combination of AZD6738 with AZD1775 showed further decrease in the number of colonies (Fig. 4, plastic, black bars, *, p < 0.05, **, p < 0.01). Effect of these drug treatments on anchorage-independent growth was also evaluated by soft agar assay (Fig. 4, soft agar). Combination treatment also further decreased number of colony formation compared to single agent treatment on soft agar (Fig. 4, soft agar, black bars, *, p < 0.05, **, p < 0.01). These results correlated with the degree of inhibition as measured by anchorage-dependent growth of OCCC on plastic (Fig. 4). At the highest concentration of both AZD6738 and AZD1775, few number of colonies were found growing in anchorage-independent soft agar (Fig. 4, soft agar,) and combination significantly reduced additional colony formation only in KOC7C (Fig. 4A, soft agar, *, p < 0.05).

**Fig. 5:**
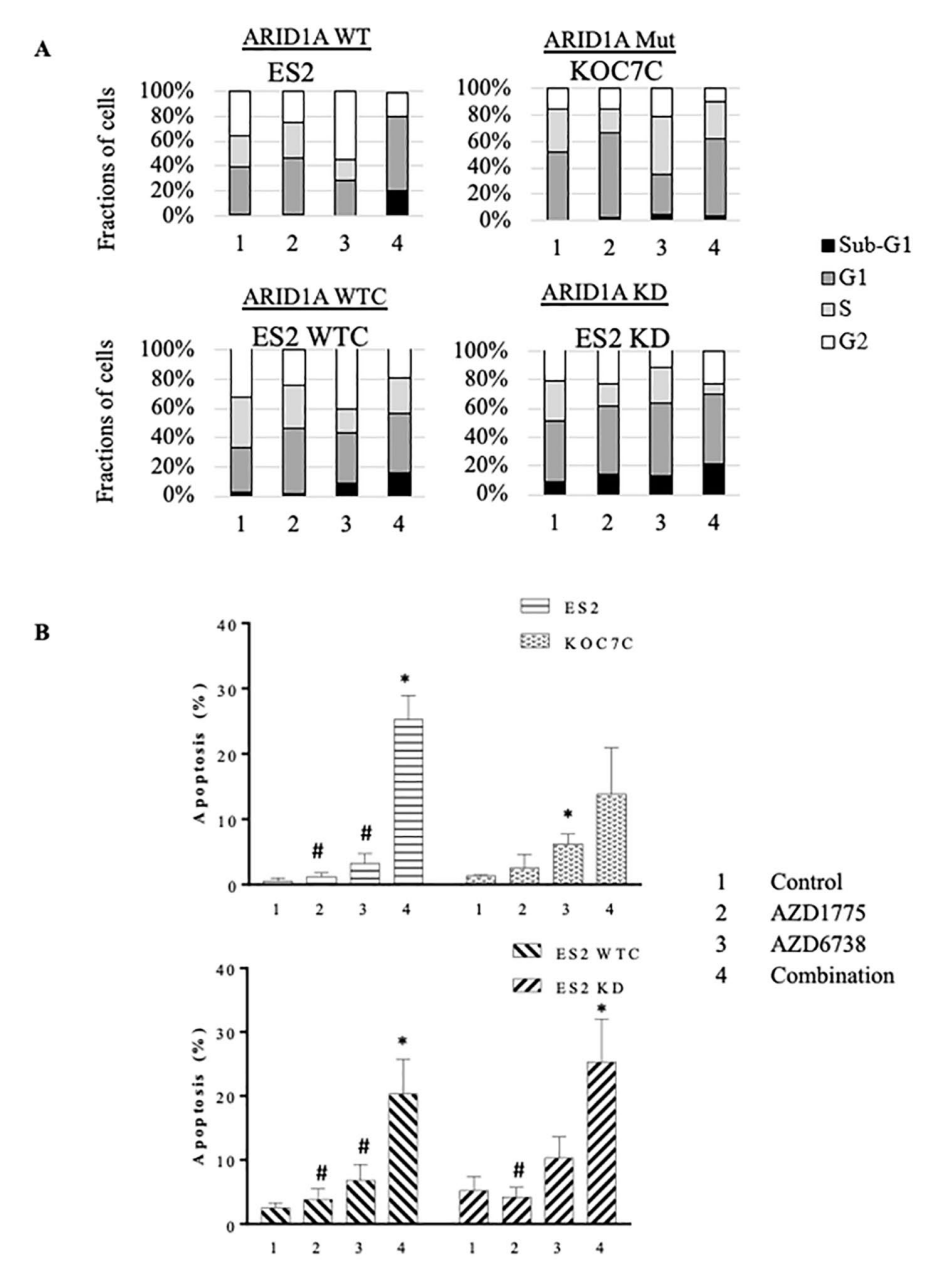
Analysis of cell cycle and apoptosis. Analysis of OCCC cell lines treated with AZD1775 and AZD6738 (WT: ES2; Mut: KOC7C; ARID1A isogenic: ES2 WTC, ES2 KD) treated with AZD1775, AZD6738 or combination of both at IC50 for 24 h. (A) Cell cycle analysis. Stacked bar graphs show the fractions of cells at Pre-G1, G1, S or G2/M phase. (B) Apoptosis analysis. Bar graphs show the fractions of annexin V positive cells. (Two-tailed t test: *, p < 0.05 compared to control; #, p < 0.05 combination compared to single agent, n = 2). Also see Supplementary Fig. 6

**Fig. 6:**
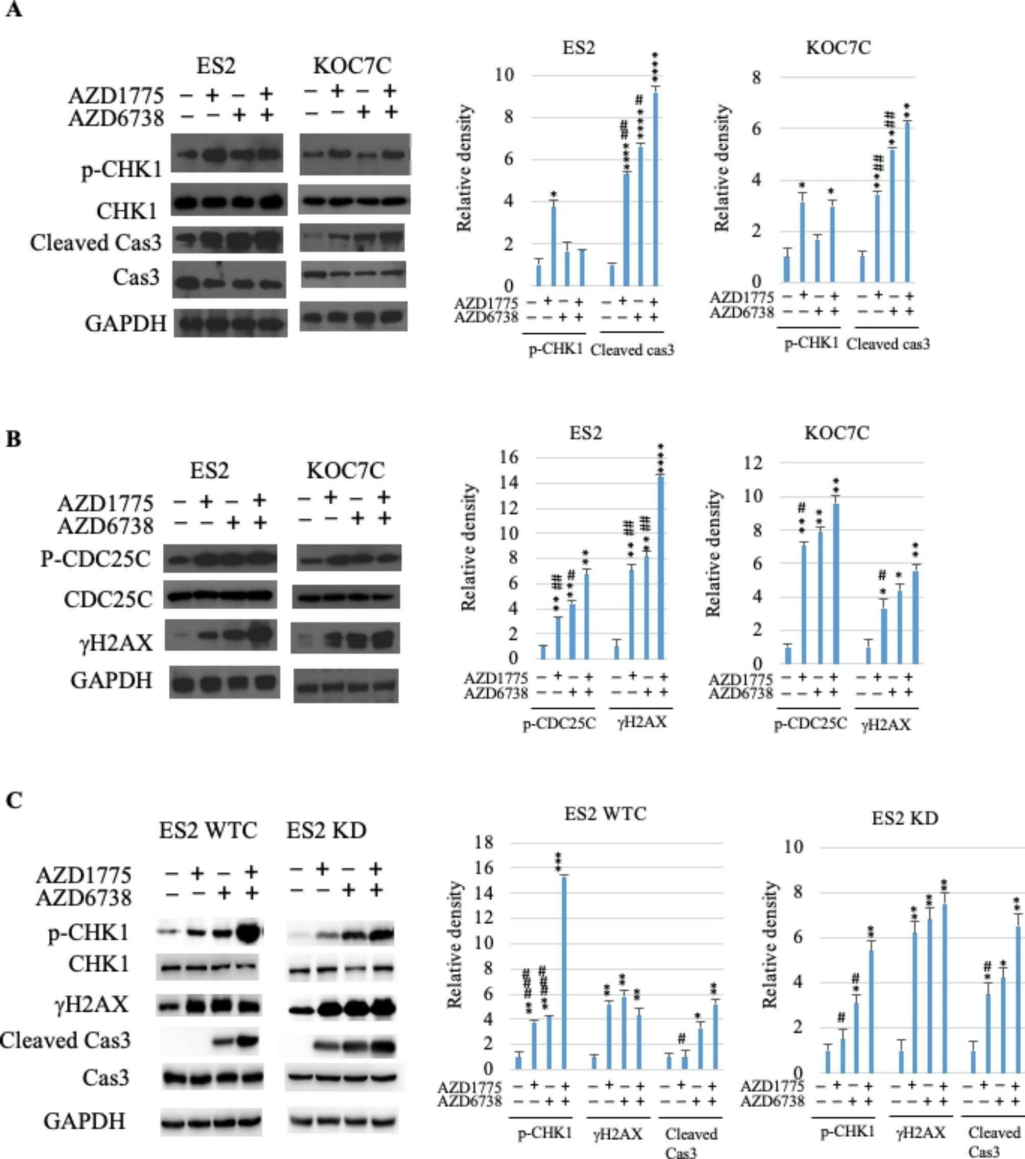
Effect of AZD1775 and AZD6738 on expression levels of protein related to apoptosis and DNA damage. A & B. Two OCCC cell lines (WT: ES; Mut: KOC7C) were treated with AZD1775, AZD6738, or combination of both and protein expression levels were examined by Western blots. C. Western blot analysis of ARID1A isogenic pair ES2 WTC, ES2 KD after drug treatment. GAPDH was used as loading control. p-CHK1, phosphorylated CHK1; Cas3, caspase 3; p-CDC25C, phosphorylated CDC25C. Bar graphs show quantification of Western blots using ImageJ as described in Methods. (Two-tailed t test: *, p < 0.05, **, p < 0.01, ****, p < 0.0001, compared to control; #, p < 0.05, ##, p < 0.01, ###, p < 0.001, combination compared to single agent; n = 2)

Effects of these drugs on cell cycle were examined in OCCC cell lines (WT: ES2, OVCA429, RMG1; Mut: KOC7C, OVTOKO, TAYA; Isogenic WTC and KD: ES2). After single agent treatment of AZD1775, OCCC cells increased in G1 phase (≤ 23%) (Fig. 5A, Supplementary. Figure 6 A). Cells treated with AZD6738 entered either G1 (≤ 24%) or G2 (≤ 19%) phase of the cell cycle (Fig. 5A, Supplementary. Figure 6 A). Treatment of cell with combination of both drugs produced prominent G1 arrest (≤ 21%) and/or increase in fragmented DNA at sub-G1 phase (≤ 40%) (Fig. 5A, Supplementary. Figure 6 A). Effect of drug treatment in apoptosis was evaluated by Annexin V staining. In WT cell lines (ES2, OVCA429, RMG1), fractions of apoptotic cells were significantly (p < 0.05) increased after combination treatment with AZD6738 and AZD1775 compared to single agent alone (Fig. 5B, Supplementary. Figure 6B). In Mut cell lines, significantly (p < 0.05) increase fractions of apoptotic cells were found after AZD6738 treatment in KOC7C (Fig. 5B) but not in OVTOKO or TAYA (Supplementary. Figure 6B). Treatment with AZD1775 showed no change in apoptotic fractions in KOC7C (Fig. 5B) or OVTOKO (Supplementary. Figure 6B) but significantly (p < 0.05) more in TAYA (Supplementary. Figure 6B). Combination treatment however did not enhance significant change in apoptotic contents in Mut cell lines. In ARID1A isogenic ES2 cells, AZD1775 or AZD6738 alone increased apoptotic cell fractions and combination treatment enhanced further apoptosis in WTC control (Fig. 5B). Treatment with AZD1775 or AZD6738 alone did not cause significant apoptosis in ES2 KD cells. Even so, combination treatment triggered substantial apoptosis in ES2 KD cells (Fig. 5B).

Protein expression levels of molecules involved in cell cycle arrest and apoptosis were measured after drug treatment. Inhibitors of either WEE1 (AZD1775) or ATR (AZD6738) alone increased levels of cleaved caspase-3 in OCCC cell lines (WT: ES2; Mut: KOC7C; ARID1A isogenic: ES2 WTC, ES2 KD) and their combination further significantly enhanced expression levels of active caspase-3 (Fig. 6A C). CHK1 is a regulator involved in cell cycle checkpoint and DNA damage response. Phosphorylation levels of CHK1 were increased after AZD1775 treatment; however, only marginal increase was found after either AZD6738 alone or the combination of AZD1775 and AZD6738 (Fig. 6A). Also, single agent of either AZD1775 or AZD6738 increased phosphorylation of CDC25C and combination of both markedly enhanced phosphorylation of CDC25C (Fig. 6B). AZD1775 or AZD6738 each alone showed increase in DNA damage (measured by levels of γH2AX). Combination of AZD1775 and AZD6738 significantly enhanced DNA damage in ES2 cells compared to each single drug alone (Fig. 6B).

## Discussion

OCCC patients have poor response to standard chemotherapy [3]. Approximately 50% of OCCC have loss of ARID1A protein expression [8, 9]; thus synthetic lethal therapy based on ARID1A deficiency has been proposed [14–16, 18, 39]. We used ARID1A isogenic cells in high throughput drug screening and identified and validated 7 drugs that are potent in killing OCCC. These include compounds targeting either mTOR (pp242), dual mTOR/PI3K (GDC0941), ATR (AZD6738), WEE1 (AZD1775), MDM2 (RG7388), dual BCL2/BCL-XL (AZD4320), or SRC (AZD0530). Analysis of the GDSC database, which contains drug response data and genomic markers of sensitivity of ~ 1,000 human cancer cell lines screened about 400 compounds and validated 4 of our drugs (AZD6738, AZD1775, GDC0941, AZD0530) which were active in a few OCCC cell lines. Several public databases such as GDSC, LINCS-L1000 [40], Cancer Therapeutics Response Portal (CTRP) [41], and Cancer Dependence Map Database (Achilles) [42] provide data of drugs’ sensitivity in cancer cell lines to help accelerate discovery of therapy for various cancers [32, 33, 43]; however, limited number of assays were assessed using OCCC cell lines. Our library of 166 compounds, only 72 have been surveyed in other types of cancer, and only 32 have been tested in OCCC. With its limitation, this enforces the significance of drug screening specifically dedicated to OCCC cells. Moreover, we have also included many other potential targets and novel inhibitors with less toxicity in OCCC.

Our hypothesis of using ARID1A isogenic OCCC cell lines which compared WTC and KD provides a more causal readout of ARID1A gene function. Each cell line has different genetic background. Nevertheless, our data was confirmed by further analyses of multiple OCCC cell lines. A prior study comparing ARID1A-mutatnt cell lines to ARID1A-wildtype cell lines used shRNA library targeting 535 human kinases and identified inhibitors of BET were more potent in ARID1A-mutant cells [17]. BET inhibitor JQ1 activity was validated in ARID1A CRISPR knockout clones from OVCA429 cell line by 6-day viability assay [17]. In our model comparing ARID1A shRNA knockdown cells (KD) to wildtype control OVCA429 cell lines, no difference in sensitivity to JQ1 while less sensitive to another BET inhibitor CPI-0610 after 3-day treatment. Another model using ARID1A isogenic RMG1 OCCC cell lines was screened with 15 small molecule inhibitors targeting epigenetic regulators and 11 shRNA targeting HDAC genes and identified both EZH2 and HDAC6 inhibitors were more sensitive in ARID1A-mutant cell lines [16, 18]. Our study found pan-HDAC inhibitor panobinostat exhibited activity in OCCC cell lines, however, independent of ARID1A status. Development of HDAC inhibitors is in advanced stage and currently there are clinical trials of HDAC inhibitors (belionostat, vorinostat, entinostat) in ovarian cancer [44–46]. To achieve high efficacy for small molecule inhibitor-based combination, robust biomarkers for selection of patients are essential. A recent study suggests KANSL1 may be a biomarker for improved survival and HDAC inhibition in ovarian cancer [47]. Applicability of this biomarker remains to be evaluated in OCCC.

One drug (AZD4320) targeting both BCL2 and BCL-XL and was selectively more active in Mut compared to WT OCCC cell lines. This inhibitor induced tumor regression and remission in venetoclax-resistant acute myeloid leukemia (AML) [48] and B-cell lymphomas [49]. BCL-XL is highly expressed in OCCC [50]; hence, targeting both BCL-XL and BCL2 may actively suppress growth of OCCC. Another drug of interest was the MDM2 inhibitor (RG7388) which was more potent in KD cells. Elevated expression of MDM2 is associated with poor prognosis in OCCC [51]. Prior studies showed other MDM2 inhibitors [including AMG [52] and RG7112 [53]], reduced OCCC cell viability. Our MDM2 inhibitor (RG7388) is in phase 3 clinical trials in patients with relapsed or refractory AML suggesting the inhibitor can quickly be tested in OCCC patients. Also, a MDM2 PROTAC (proteolysis targeting chimera) has been developed [54] and should be tested against OCCC.

Activated SRC is associated with an unfavorable survival in endometriosis-associated OCCC [55]. In our study, the SRC inhibitors (saracatinib, bosutinib, dasatinib) had higher anti-proliferative activity in KD compared to WTC. Dasatinib had anti-tumor activity against OCCC [56]. Likewise, a previous drug screening of 68 compounds identified dasatinib as more active in ARID1A mutant OCCC compared to ARID1A wild type OCCC [57]. Although single agent dasatinib has minimal activity in ovarian cancer clinical trial [58], a current trial assessing clinical activity of dasatinib includes OCCC patients and their ARID1A expression status (NCT02059265). Another study suggests PTTG1 expression levels may be a biomarker for prediction of sensitivity to saracatinib in all types of ovarian cancer [59]. Saracatinib should be investigated in OCCC.

Another frequently activated kinase is PIK3CA (activation mutations found in approximately 50% of OCCC). A prior study identified mTORC1/2 inhibition as treatment for OCCC by analysis of kinase mutations (518 kinases) and copy number alterations [20]. We identified the dual mTOR/PI3K inhibitor (GDC0941) with anti-proliferative activity in OCCC cell lines; Also, an inhibitor of mTOR (pp242) was more potent in KD compared to WTC. Association of ARID1A mutation and PIK3CA mutation occurs [60–63]. Furthermore, a weak inhibitor of mTOR (Temsirolimus) was evaluated in phase 2 trial in combination with carboplatin and paclitaxel for advanced OCCC while no statistical improvement in progression-free survival occurred [64]. Nevertheless, our test of the more potent dual inhibitor of mTOR/PI3K (GDC0941) was very potent against OCCC cell lines.

Mutations or copy number alterations in DNA repair/cell cycle arrest/apoptosis occur in 82% of OCCC samples [20]. DNA damage response signaling is regulated by ATM, ATR, and WEE1. WEE1 also inhibits CDK1/2 activation and regulates G2/M transition. A prior high-throughput screening identified cell cycle modulators selectively targeting ARID1A-deficient cells [65]. Previous studies showed that the WEE1 inhibitor (AZD1775) enhanced carboplatin efficacy in TP53-mutated ovarian cancer in a phase 2 study [66]. However, TP53 mutation is mutually exclusive with ARID1A and PI3K mutations in OCCC tumors [20, 67]. ATR inhibitor (AZD6738) is active in ATM-deficient cells [68]; ~9% OCCC have ATM deletion mutations [20]. A previous study found synthetic lethality of ATR inhibitors in ARID1A deficient tumors [69]. Additionally, the combination of AZD6738 (ATR inhibitor) and olaparib (inhibitor of PARP (Poly ADP-ribose polymerase)) reduced tumor burden in BRCA-mutant high grade serous ovarian cancer [70] although other study showed ARID1A loss is associated with PARP inhibitor resistance [71]. Currently, a phase 2 trial of AZD6738 with combination of olaparib is ongoing for relapsed gynecological cancers [72]. Its success may allow the use of the drug in OCCC patients. We found synergy between WEE1 and ATR inhibition not only in ARID1A mutant OCCC cell lines but also in ARID1A wildtype OCCC cell lines. These cell lines all displayed increase in G1 or G2 growth arrest plus apoptosis after combination treatment; thus, ARID1A status may not be a predictor of therapeutic effect by this combination. Previously predictive biomarkers have been identified for inhibitors of WEE1 or ATR [73–76]. Combination treatment of AZD1775 and AZD6738 increases DNA damage and interferes with DNA replication. Clinical biomarkers for DNA replication stress are needed to aid in prediction of response. A recent study suggest higher copy number of CCNE1 predicts higher sensitivities of high grade serous ovarian cancer to combination of WEE1 and ATR1 [77]. Whereas, synergy effect in the combination of ATR inhibitor and WEE1 inhibitor is found in both high CCNE1-expressing osteosarcoma cell line and low CCNE1-expressing lung cancer cell lines [78]. Prior studies show ~ 20% OCCC had CCNE1 copy number gain and overexpression and are associated with poor prognosis [79, 80]. It remains to be assessed if CCNE1 as a biomarker for WEE1 and ATR1 combination is applicable to OCCC. Potentially additional markers of response are needed. These include predictive markers for WEE1 or ATR inhibitors (cdc25A, ATM, p21, RRM2, γH2AX) and common replication stress-induced FANCD2 foci formation. In addition to promotion of DNA damage and cell death, inhibitor targeting proteins in DNA repair mechanism such as ATR and WEE1 also induce anti-tumor immune responses and sensitizes cancer cells to immunotherapy [81–83]. Several ongoing clinical trials are testing inhibitors of DNA damage response molecules including ATR or WEE1 (NCT04095273, NCT05396833, NCT04158336).

## Conclusions

OCCC has low response rates versus standard ovarian cancer therapy. High throughput drug screening of ARID1A isogenic OCCC cell lines with 166 compounds (FDA-approved, or either in clinical trials or pre-clinical studies) identified 7 inhibitors targeting BH3 domain (AZD4320), WEE1 (AZD1775), mTOR (PP242), dual mTOR/PI3K (GDC0941), ATR (AZD6738), MDM2 (RG7388), or SRC (AZD0530). We further revealed combination of AZD6738 and AZD1775 synergistically induced growth inhibition of OCCC cells. These two drugs are already in other clinical trials, making them ideal candidates for treatment of OCCC. These results highlighted drug screening will contribute to discovery of novel therapeutic targets and alternative treatments for the improvement of OCCC patient outcomes.

## Electronic supplementary material

Below is the link to the electronic supplementary material.

Supplementary Material 1

## Data Availability

Not applicable.

## Abbreviations

ATR: Ataxia-telangiectasia mutated and Rad3-related
OCCC: Ovarian clear cell carcinoma
WT: Wild type ARID1A
Mut: Mutant ARID1A
WTC: Wildtype control
KD: ARID1A shRNA knockdown.
