# Supplementary material for: Treatment for ovarian clear cell carcinoma with combined inhibition of WEE1 and ATR
Source: J Ovarian Res. 2023 Apr 22;16:80. doi: 10.1186/s13048-023-01160-y (PMC10122390; doi:10.1186/s13048-023-01160-y)
Supplement: Supplementary file 1 — Supplementary Material 1 [file 13048_2023_1160_MOESM1_ESM.pdf]

Supplementary Table 1

Single agent

17-AAG (Tanespimycin)	CPI-0610	KU-55933
A-674563	Crenolanib	KW-2449
Acalabrutinib	Crizotinib (PF-2341066)	Lapatinib
Afatinib (BIBW-2992)	CYT387	Lenalidomide
Alisertib (MLN8237)	Cytarabine	Lenvatinib
AT-101	Dasatinib	Linifanib (ABT-869)
AT7519	DBZ	Masitinib (AB-1010)
Axitinib (AG-013736)	Doramapimod (BIRB 796)	Metformin
AZ3202	Dovitinib (CHIR-258)	MGCD-265
Azacytidine	Elesclomol	Midostaurin
AZD0156 (ATM)	Entospletinib (GS-9973)	MK-2206
AZD1480	Entrectinib	ML111
AZD1775 (Wee1)	Erlotinib	MLN120B
AZD2014 (mTORC)	Etomoxir	MLN8054
AZD2811(Aurora)	Flavopiridol	Motesanib (AMG-706)
AZD4320	Foretinib (XL880)	Neratinib (HKI-272)
AZD5991 (MCL1)	GDC-0575	NF-kB Activation Inhibitor
AZD6378 (ATR)	GDC-0879	Nilotinib
Baiclein	GDC-0941	Nutlin 3a
Barasertib (AZD1152-HQPA)	GDC-0994	NVP-ADW742
Bay 11-7085	Gefitinib	NVP-AEW541
BETi (TEN-010)	Gilteritinib	NVP-TAE684
BEZ235	GSK-1838705A	Olaparib
Birinapant	GSK-1904529A	OTX-015
BKH-I-157-1	GSK-2879552	Palbociclib
BKH-I-158-1	GSK690693	Panobinostat
BKH-I-158-2	GW-2580	Pazopanib (GW786034)
BKH-I-161-1	Ibrutinib (PCI-32765)	PBD (SG3199)
BLZ945	Idasanutlin	PD173955
BMS-345541	Idelalisib	Pelitinib (EKB-569)
BMS-754807	Imatinib	Pemigatinib
Bortezomib (Velcade)	Indisulam	Perhexiline maleate
Bosutinib (SKI-606)	INK-128	PH-797804
Brigatinib (AP-26113)	Ipatasertib (GDC-0068)	PHA-665752
Cabozantinib	ITK-413A	PHT-427
Canertinib (CI-1033)	ITK-413B	PI-103
Cediranib (AZD2171)	JAK Inhibitor I	Ponatinib (AP24534)
CG-026806	JNJ-38877605	PP242
CHIR-99021	JNJ-527	PRT062607
CI-1040 (PD184352)	JNJ-7706621	PT-1171-012
Cobimetinib (GDC-0973)	JQ1	PT-1857-002

Single agent (Supp. Table 1 continued)

PT-1881-002
PT-1900-003
Quizartinib (AC220)
RAF265 (CHIR-265)
Ralimetinib (LY2228820)
Ranolazine
Rapamycin
Regorafenib (BAY 73-4506)
Roscovitine (CYC-202)
Ruxolitinib (INCB018424)
Saracatinib (AZD0530)
SB-431542
SCH-772984
Selinexor
Selumetinib (AZD6244)
SGX-523
SNS-032 (BMS-387032)
Sorafenib
SR9011
Sunitinib
SY-1365
TAK-659
TAK-MLN4924
TAK-MLN7243
TAK-MLN9708
Taselisib (GDC-0032)
TG100-115
Tivozanib (AV-951)
Tofacitinib (CP-690550)
Tozasertib (VX-680)
Trametinib (GSK1120212)
UNC 2025A
Vandetanib (ZD6474)
Vargetef
Vatalanib (PTK787)
Vemurafenib (PLX-4032)
Venetoclax
Vismodegib (GDC-0449)
Volasertib (BI-6727)
VX-745
XAV-939
XMD 8-87
YM-155

Combination (Supp. Table 1 continued)

Acalabrutinib - AZD4320
Acalabrutinib - AZD5991
Azacytidine - Venetoclax
AZD6378 (ATR) - Cytarabine
BETi (TEN-010) - Venetoclax
Bortezomib - AT-101
Bortezomib - Birinapant
Bortezomib - GSK2879552
Bortezomib - OTX-015
Bortezomib - Palbociclib
Bortezomib - Venetoclax
CG-026806 - OTX-015
CG-026806 - Venetoclax
Doramapimod - AT-101
Doramapimod - Birinapant
Doramapimod - GSK2879552
Doramapimod - OTX-015
Doramapimod - Palbociclib
Doramapimod - Venetoclax
GW-2580 - AT-101
GW-2580 - Birinapant
GW-2580 - GSK2879552
GW-2580 - OTX-015
GW-2580 - Palbociclib
GW-2580 - Venetoclax
Idelalisib - AT-101
Idelalisib - Birinapant
Idelalisib - GSK2879552
Idelalisib - OTX-015
Idelalisib - Palbociclib
Idelalisib - Venetoclax
Olaparib - AT-101
Olaparib - Birinapant
Olaparib - GSK2879552
Olaparib - OTX-015
Olaparib - Palbociclib
Olaparib - Trametinib
Olaparib - Venetoclax
Quizartinib - AT-101
Quizartinib - Birinapant
Quizartinib - GSK2879552
Quizartinib - OTX-015
Quizartinib - Palbociclib
Ruxolitinib - AT-101
Ruxolitinib - Birinapant
Ruxolitinib - GSK2879552
Ruxolitinib - OTX-015
Ruxolitinib - Palbociclib
Ruxolitinib - Venetoclax
Taselisib - Venetoclax
Trametinib - AT-101
Trametinib - Birinapant
Trametinib - GSK2879552
Trametinib - OTX-015
Trametinib - Palbociclib
Venetoclax - Ibrutinib
Venetoclax - PH797804

Supplementary Table 2

	shC Rep 1	shC Rep 2	sh1 Rep 1	sh1 Rep 2
17-AAG	0.0307	0.0314	0.0293	0.0255
Alisertib	0.4628	0.3396	0.1513	0.1740
AZD0156	1.0000	1.0000	0.0556	1.0000
AZD1480	10.0000	10.0000	5.9834	3.9740
AZD1775	0.1669	0.1796	0.0867	0.0738
AZD2014	0.5199	0.3577	0.0641	0.2733
AZD2811	0.4877	0.6372	0.1188	0.2771
AZD6378	2.8195	2.4159	0.3466	1.3457
BMS-754807	5.4250	5.9307	3.8349	4.5781
Bosutinib	5.6915	6.8873	4.7507	4.1164
CHIR-99021	7.2578	6.6392	8.0768	7.9740
CI-1040	10.0441	9.3199	7.6800	8.2850
Cobimetinib	2.8369	2.8050	0.7642	0.9643
CPI-0610	5.1319	5.0457	10.0000	10.0000
Cytarabine	0.0666	0.0739	0.0885	0.0974
Dasatinib	0.0419	0.0518	0.0264	0.0227
Dovitinib	3.2652	3.4425	2.8516	2.6520
Etomoxir	6.5215	7.7452	11.5554	11.9259
GDC-0575	0.2280	0.2225	0.1284	0.0792
Idasanutlin	0.1775	0.1620	0.1111	0.0607
Lenvatinib	10.0000	10.0000	5.9049	6.4490
ML111	1.0101	0.9496	2.6070	2.5974
MLN8054	1.5571	2.1491	0.8037	1.0268
Pelitinib	0.3213	0.3387	0.2878	0.2650
Pemigatinib	5.6550	4.3844	2.6155	2.8786
PP242	0.4772	1.4062	0.2811	0.0186
Saracatinib	10.0000	7.1952	3.8053	4.6656
Selinexor	0.0611	0.0569	0.0189	0.0180
Selumetinib	10.0000	10.0000	5.0032	6.9964
SNS-032	0.1256	0.1291	0.0991	0.0886
Sunitinib	0.9059	0.8649	1.0676	1.0286
Taselisib	1.6017	1.3603	0.3911	0.2406
Tozasertib	0.9725	0.8217	0.5795	0.4985
UNC 2025A	0.6333	0.4937	0.2174	0.1137
Vemurafenib	7.2252	7.2611	8.3749	8.4155
Volasertib	0.2271	0.0942	0.0154	0.0852
XAV-939	8.9937	8.8726	8.3626	8.6132
YM-155	0.0015	0.0018	0.0026	0.0023

IC50 values (μM) of OVCA429 cell lines (shC: control wild type OCCC; sh1: ARID1A knockdown; Rep 1, Rep 2: biological duplicates) treated for 72 hr.

Supplementary Table 3

	shC Rep 1	shC Rep 2	sh1 Rep 1	sh1 Rep 2
Bortezomib	179.4	245.3	218.4	202.7
Canertinib (CI-1033)	3879.4	4664.0	3714.8	3936.4
Elesclomol	9.1	10.0	9.3	11.3
Erlotinib	7759.9	8290.4	6225.8	6798.4
Flavopiridol	77.7	58.6	24.3	70.4
Indisulam	223.2	2199.3	1057.0	311.9
INK-128	9.1	30.2	20.6	9.4
Midostaurin	454.1	796.6	280.6	474.5
Nutlin 3a	1880.6	3093.8	2005.9	2482.0
Palbociclib	2481.3	6487.9	1937.4	4966.9
Panobinostat	18.1	25.4	19.4	28.8
PBD (SG3199)	0.1	0.1	0.1	0.1
PI-103	119.3	161.7	123.2	99.4
RAF265 (CHIR-265)	187.1	266.6	239.9	208.2
Rapamycin	19.8	107.6	17.8	27.4
SCH-772984	842.4	394.7	174.7	27.7
SY-1365	8.8	8.8	8.7	8.5
TAK-MLN4924	501.6	567.7	561.9	567.8

IC50 values (nM) of OVCA429 cell lines (shC: control wild type OCCC; sh1: ARID1A knockdown; Rep 1, Rep 2: biological duplicates) treated for 72 hr.

Supplementary Table 4

Combinations		shC Rep 1	shC Rep 2	sh1 Rep 1	sh1 Rep 1	*p* value
Acalabrutinib	AZD4320	10.00	7.75	4.50	1.78	0.04339
Bortezomib	AT-101	5.38	4.11	0.18	0.15	0.04372
CG-026806	Venetoclax	0.34	0.50	0.01	0.15	0.04325
GW-2580	OTX-015	1.27	0.44	2.79	3.44	0.02770
Quizartinib	AT-101	6.40	6.64	9.89	9.23	0.02170
Ruxolitinib	Palbociclib	10.00	10.00	2.14	5.45	0.08293
Trametinib	AT-101	0.52	0.50	0.07	0.02	0.00831
Trametinib	GSK2879552	10.00	10.00	0.04	0.02	0.00032
Trametinib	Palbociclib	0.11	0.14	0.03	0.02	0.03698

IC50 values (mM) of OVCA429 OCCC cell lines (shC: control wild type OCCC; sh1: ARID1A knockdown; Rep 1, Rep 2: biological duplicates) after 72 hr treatment of two drug combinations. *P* value: *t* test (one - tailed) comparison between WTC [shC] and KD [sh1].

Supplementary Table 5

In-house collection of cell lines
at National University Cancer Institute of Singapore

Name	Media	Source
JHOC5	RPMI + 10% FBS	RIKEN
ES2	RPMI + 10% FBS	ATCC
OVCA429	DMEM + 10% FBS	NUS
RMG1	RPMI + 10% FBS	JCRB
OVTOKO	RPMI + 10% FBS	JCRB
KOC7C	RPMI + 10% FBS	NUS
RMG2	RPMI + 10% FBS	JCRB
TAYA	RPMI + 10% FBS	NUS
JHOC9	RPMI + 10% FBS	RIKEN
RMG5	RPMI + 10% FBS	JCRB

Abbreviations:
RIKEN: RIKEN BioResource ResearchCenter
ATCC: American Type Culture Collection
JCRB: Japanese Collection of Research Bioresources Cell Bank
NUS: National University of Singapore

Supplementary Figure Legends.

Supplementary Figure 1 ARID1A expression analysis in OCCC cell lines. A. Protein expression levels of ARID1A in 9 OCCC cell lines (4 ARID1A wild-type and 5 ARID1A mutant) were analyzed by Western blotting. *, ARID1A mutant. B. Knockdown of ARID1A in 3 cell lines were verified by real-time PCR (bar graphs) and Western blot analysis.

Supp. Figure 2. Identification of small molecules with anti-tumor activity in ARID1A wild type and mutant OCCC cell lines. MTT assay of 12 inhibitors: mTOR (PP242), dual mTOR/PI3K (GD0941), ATR (AZD6738), WEE1 (AZD1775), ATM (AZD0156), MERTK (UNC2025), UAE (MLN7243), AURKB (AZD1152), MDM2 (RG7388), MEK1 (GD0973), BH3 (AZD4320), and SRC (AZD0530) for 7 OCCC cell lines (4 WT: ARID1A wild type, JHOC5, ES2, OVCA429, RMG1; 2 Mut: ARID1A mutant, KOC7C, OVTOKO). Cells were treated with these compounds for 72 hr.

Supp. Figure 3. IC50 values of inhibitors of ATR (AZD6738), WEE1 (AZD1775), mTOR/PI3K (GD0941) and SRC (AZD0530) in GDSC database. Scattered dot plots of IC50s of 4 drugs (AZD6738, AZD1775, GD0941, AZD0530) from GDSC are shown. Red bar, Geometric mean of IC50.

Supp. Figure 4. Combination treatment between AZD1775 and AZD6738 in OCCC cell lines. A. ARID1A isogenic JOHC5 OCCC cell lines (WTC: wildtype control; KD: ARID1A shRNA knockdown). B. OCCC cell lines (WT: OVCA429, RMG1,; Mut: OVTOKO, TAYA) were treated with AZD1775 or AZD6738 alone and combination of both at 0, 0.25, 0.5, 1, 2 μM for 72 h. Dose-response matrices and CI (combination index) heatmap between the interaction of two drugs are shown. Dose-response matrices: Red; 100% growth inhibition; White; 50%; Blue, 0%. CI's were analyzed by CompuSyn as described in Methods. CI values: < 1, synergistic; =1, additive; > 1, antagonistic. CI < 0.3, strong synergy.

Supp. Figure 5. Synergy analysis on the combination treatment of AZD1775 and AZD6738 in OCCC cells. OCCC cell lines (ARID1A wild type: RMG1, OVCA429, ES2; ARID1A mutant: KOC7C, OVTOKO, TAYA) were treated with AZD1775 or AZD6738 alone and combination of both at 0, 0.25, 0.5, 1, 2 μM (< IC50) for 72 h. Synergy maps between the interaction of two drugs were analyzed by SynergyFinder 2.0. HSA model were tested as described in Materials & Methods

and scores for each cell line are shown in table. Score represents likely hood between two drugs: HSA scores < -10, likely antagonistic; -10 < score < 10, likely additive; score > 10, likely synergistic.

Supp. Figure 6. Cell cycle progression and apoptosis analysis of AZD1775 and AZD6738 treatment in OCCC cell lines. OCCC cell lines (WT: ARID1A wild type: OVCA429, RMG1; Mut: ARID1A mutant: OVTOKO, TAYA) treated with AZD1775, AZD6738 or combination of both at IC50 for 24 h. A. Cell cycle analysis. Stacked bar graphs show the fractions of cells at Pre-G1, G1, S or G2/M phase. B. Annexin V assay analysis. Bar graphs showed annexin V-positive fractions of cells after drug treatment. Representative results are shown from 2 independent experiments. Two-tailed t test was performed. *, $p < 0.05$, compared to control; #, $p < 0.05$ combination treatment compared to single agent.

A

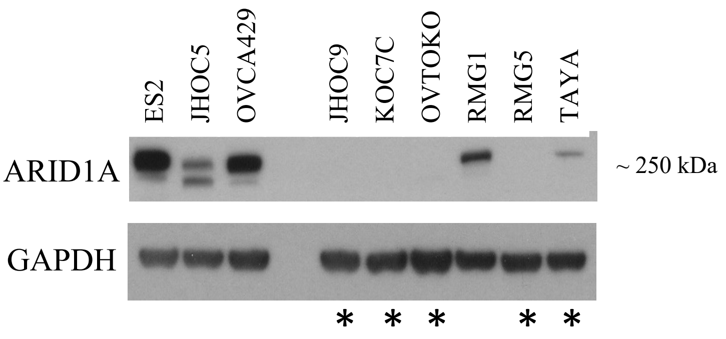

B

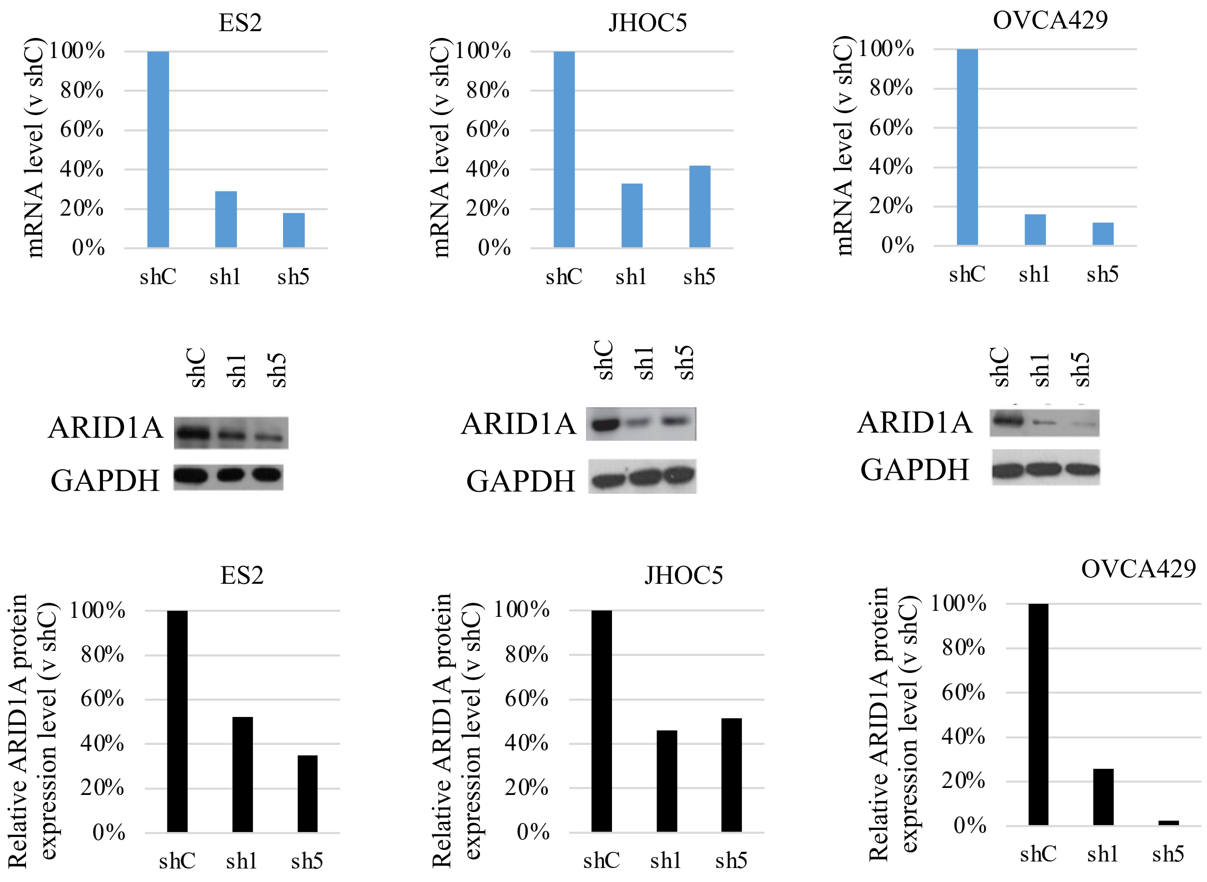

Supplementary. Fig. 1

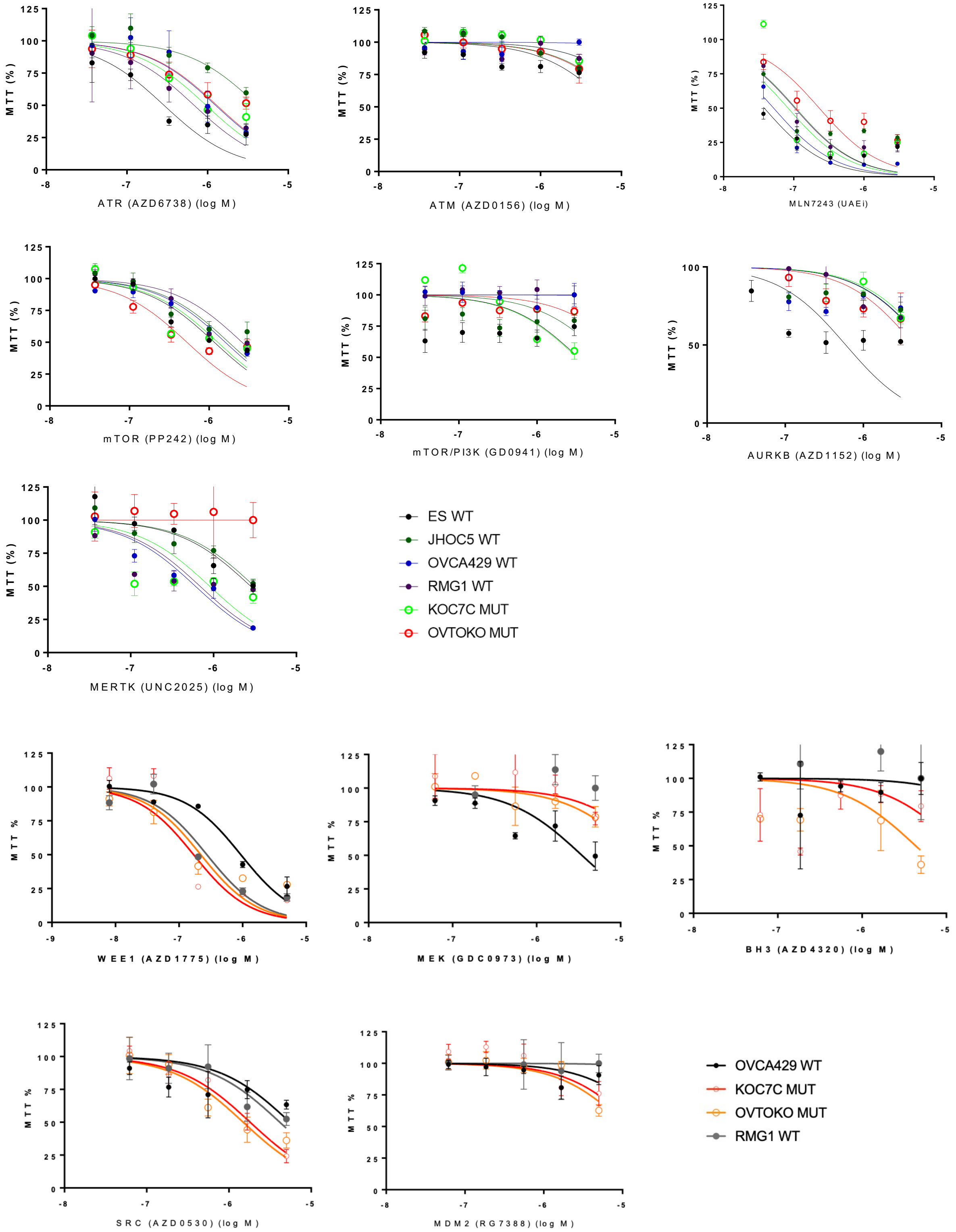

Supplementary Fig. 2

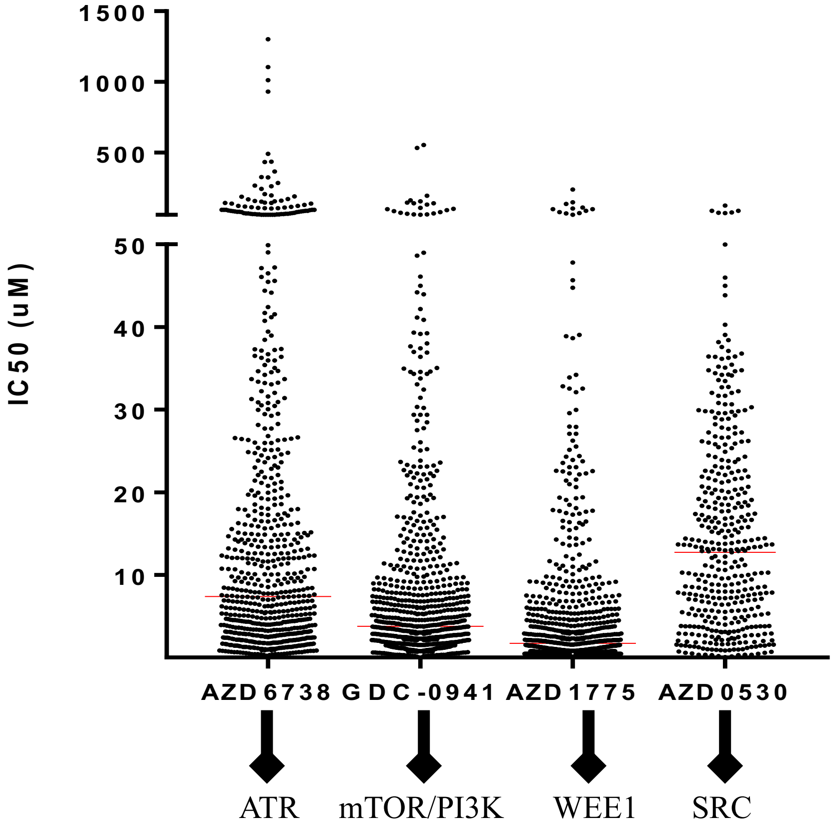

OCCC cell lines	IC50* (μM)			
	AZD6738	GDC-0941	AZD1775	AZD0530
ES2	4.86	19.33	0.91	7.41
EFO21	145.56	6.91	4.38	na
RMG1	13.17	3.97	3.61	na
OC314	3.39	1.39	0.82	na
OVISE	7.04	3.00	1.65	na
OVTOKO	21.73	1.12	3.38	na
TOV21G	2.59	5.50	0.68	na
Geometric mean of IC50 (n = number of cell lines tested in GDSC)	8.8 (n = 776)	4.2 (n = 766)	1.9 (n = 770)	9.2 (n = 408)
*IC50 values were downloaded from GDSC.				

Supplementary Fig. 3

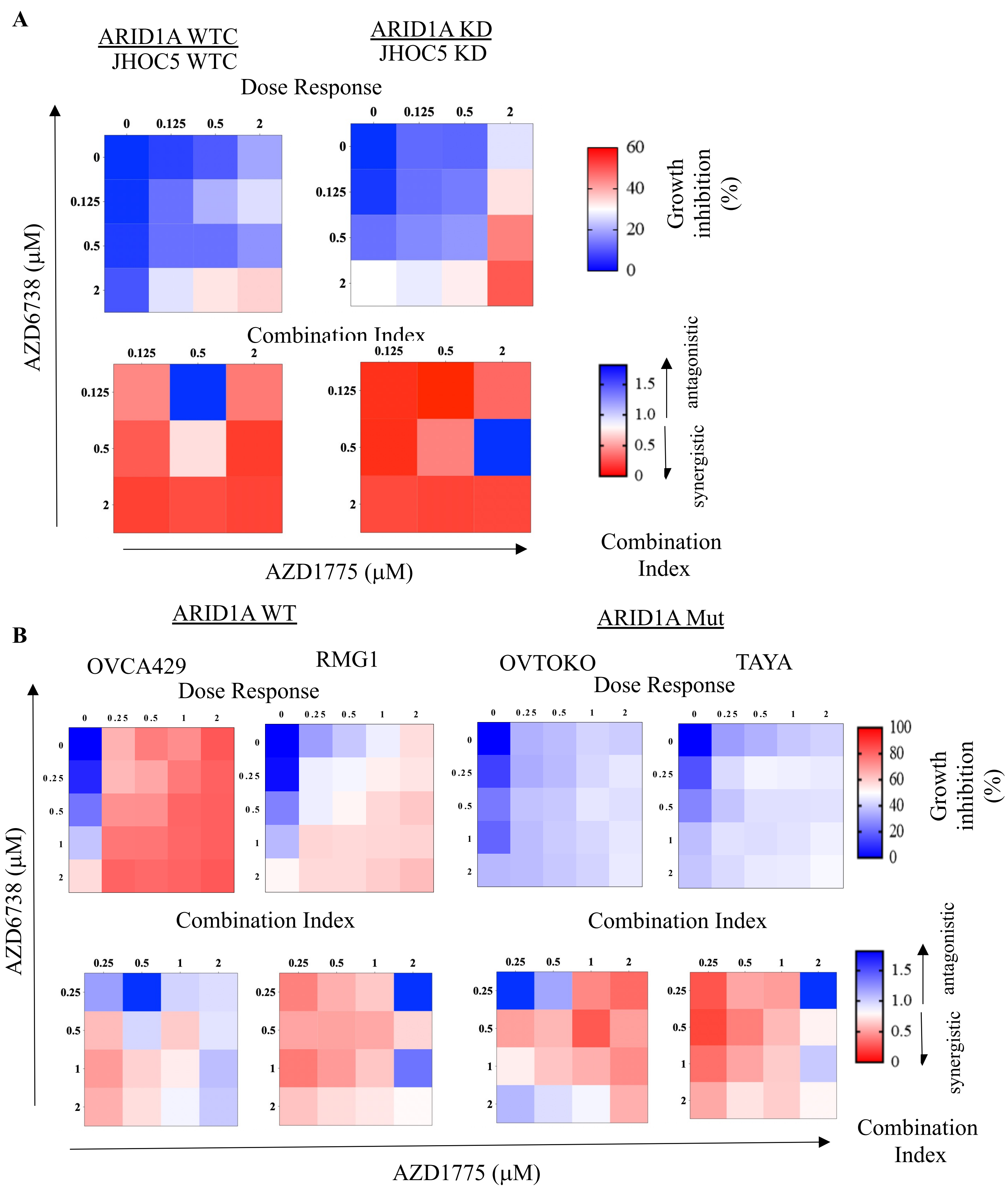

Supplementary Fig. 4

ARID1A Wild type

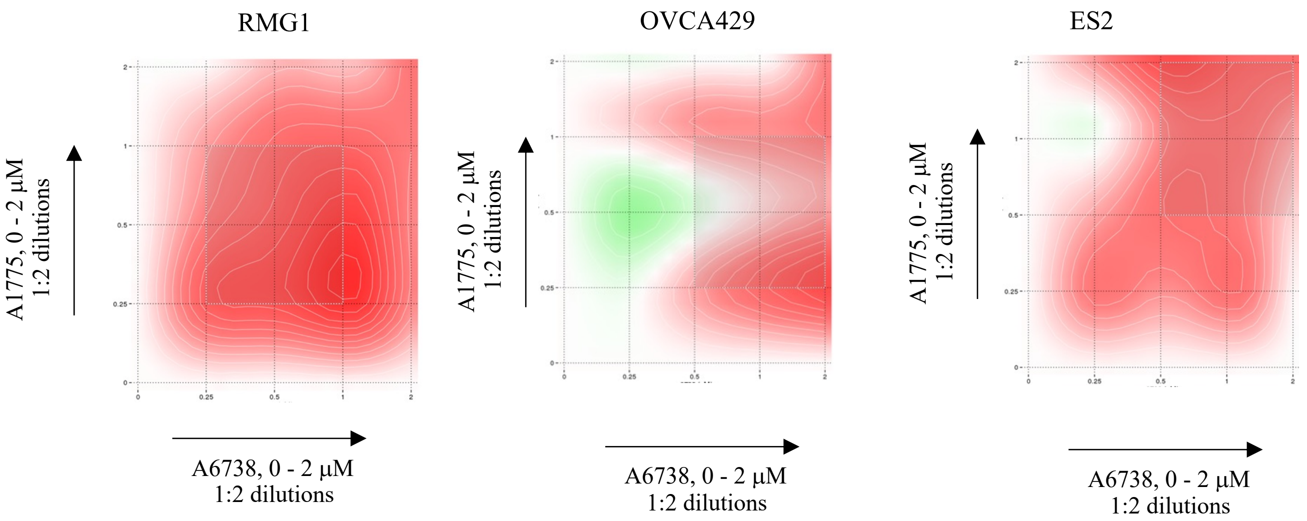

ARID1A Mutant

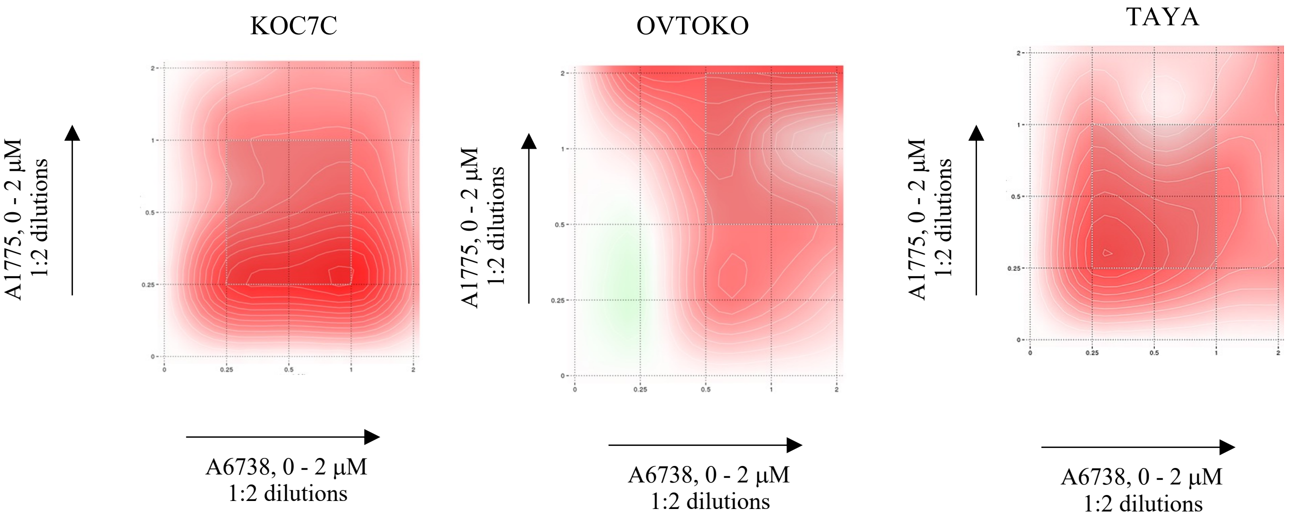

AZD1775 and AZD6738 combination HSA score		
ARID1A wild type	RMG1	11.5
	OVCA429	4.4
	ES2	9.1
ARID1A mutant	KOC7C	11.3
	OVTOKO	2.7
	TAYA	8.6

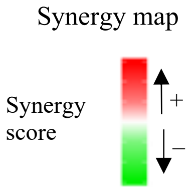

Supplementary Fig. 5

A

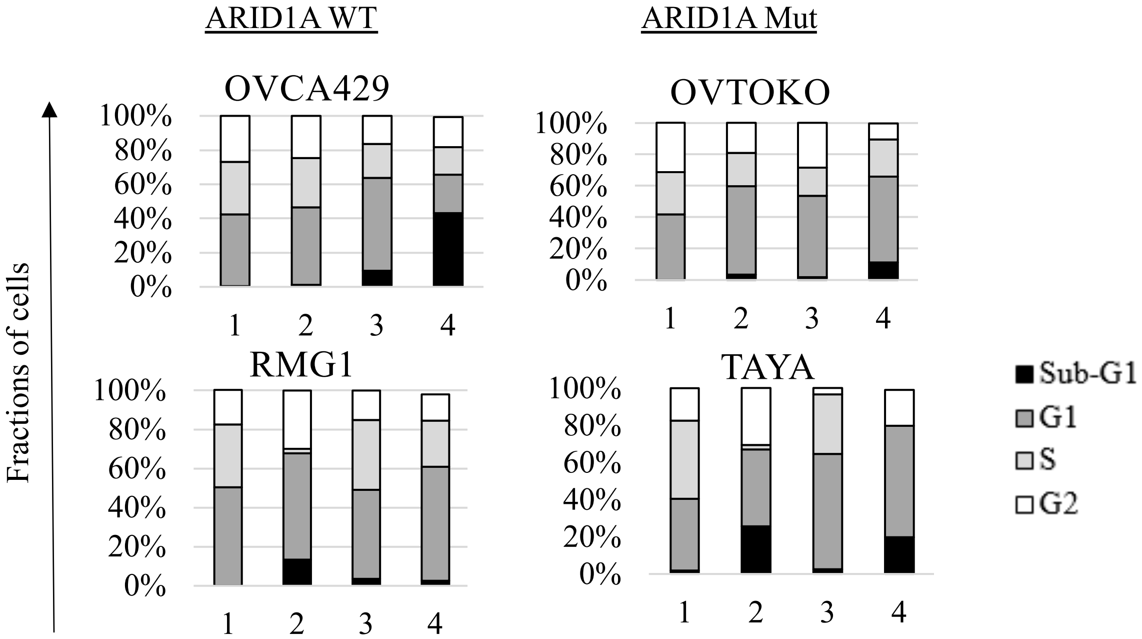

B

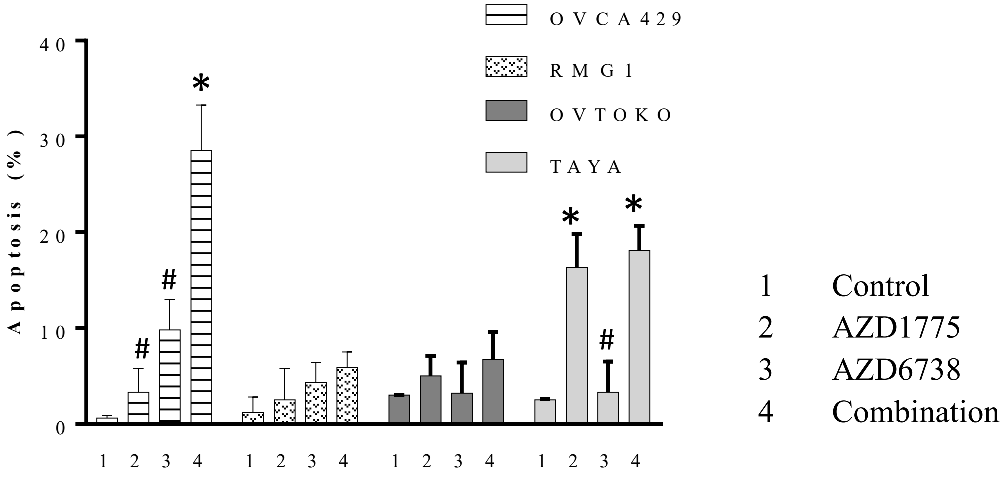

Supplementary Fig. 6
